# Corrosion in Reinforced Concrete Panels: Wireless Monitoring and Wavelet-Based Analysis

**DOI:** 10.3390/s140203395

**Published:** 2014-02-19

**Authors:** Guofu Qiao, Guodong Sun, Yi Hong, Tiejun Liu, Xinchun Guan

**Affiliations:** 1 Key Lab of Structures Dynamic Behavior and Control, Harbin Institute of Technology, Ministry of Education, Harbin 150090, China; E-Mail: guanxch@hit.edu.cn; 2 School of Civil Engineering, Harbin Institute of Technology, Harbin 150090, China; 3 School of Information Science and Technology, Beijing Forestry University, Beijing 100083, China; 4 Centre for Composite Material and Structure, Harbin Institute of Technology, Harbin 150090, China; E-Mail: hongyi@hit.edu.cn; 5 Shenzhen Graduate School, Harbin Institute of Technology, Shenzhen 518055, China; E-Mail: liutiejun@hit.edu.cn

**Keywords:** reinforced concrete structures, corrosion monitoring, wireless sensor and networks, electrochemical emission, wavelet

## Abstract

To realize the efficient data capture and accurate analysis of pitting corrosion of the reinforced concrete (RC) structures, we first design and implement a wireless sensor and network (WSN) to monitor the pitting corrosion of RC panels, and then, we propose a wavelet-based algorithm to analyze the corrosion state with the corrosion data collected by the wireless platform. We design a novel pitting corrosion-detecting mote and a communication protocol such that the monitoring platform can sample the electrochemical emission signals of corrosion process with a configured period, and send these signals to a central computer for the analysis. The proposed algorithm, based on the wavelet domain analysis, returns the energy distribution of the electrochemical emission data, from which close observation and understanding can be further achieved. We also conducted test-bed experiments based on RC panels. The results verify the feasibility and efficiency of the proposed WSN system and algorithms.

## 1. Introduction

Corrosion of reinforcing steel, generally caused by carbonation or Cl^−^, is the most important factor causing severe degradation of the durability of reinforced concrete (RC) structures. Especially, Cl^−^ from the concrete itself or the environment leads to pitting corrosion which will quickly result in a remarkable reduction of the cross section of the reinforcing steel [1,2]. The durability deterioration of RC structures finally leads to very high repair costs, sometimes much greater than the initial construction cost, and in some extreme situations, can result in the collapse of the structure [3]. With the development of global warming and further deterioration of the environment, the service condition of RC structures has recently become much harsher.

Over the past two decades, structural health monitoring (SHM) has gained worldwide acceptance as an affordable way to obtain real-time data on the health, and consequently, on the safety and the serviceability of civil infrastructure systems [4]. There have been many sensing technologies applied in earthquake engineering, wind engineering and life-cycle performance evaluation, such as the fiber optic sensing technique [5], the wave propagation-based piezoelectric ceramic (PZT) sensing technique [6–9] and the smart cement-based sensing technique, *etc.* [10,11]. Recently, future trends in the development of sensing technologies and SHM in civil engineering have been critically reviewed and put forward [12–14].

Considering the severity of the reinforcing steel's corrosion, it is very urgent to recognize the corrosion status of the steel bar, and then to provide early warning information before the RC structures reach an unsafe condition in the field. Unfortunately, although the corrosion mechanism of reinforcing steel has been investigated extensively in the past few decades, the corrosion monitoring of RC structures has still not been well addressed till now. Some significant challenges related to the corrosion monitoring methods and sensors, therefore, should be carefully considered and seriously clarified for the actual SHM systems. Firstly, the purpose of corrosion monitoring is to actively idey the corrosion status according to the electrochemical characteristics of RC structures. In contrast to the traditional works implemented by most researchers in laboratories, corrosion monitoring is the inverse process of the electrochemical characterization of RC structures. Secondly, the evolution of the reinforcing steel's corrosion will be influenced by the environmental conditions over time. Therefore, *in-situ*, real-time and on-line ideication of the corrosion status is another key issue. Furthermore, the foregoing engineering practice indicates that the distribution of large amounts of conducting wires, demanding massive labor and financial resources represent annoying problems in SHM for civil engineering.

The pitting corrosion, known as “steel cancer”, significantly deteriorates the durability of RC structures and greatly degrades their serviceability. The electrochemical emission spectrum (EES) can be used as the most intrinsic feature of the pitting corrosion caused by Cl^−^ [15]. Electrochemical emissions, generally considered as the “fingerprints” of pitting corrosion, reflect the information of the initial, metastable, repassivation and stable stages during the corrosion process [16,17]. Therefore, the pitting corrosion status could be ideied effectively via this intrinsic information by passively listening to the reinforcing steel to determine how the corrosive media intrudes into the RC structures [18–21]. On the other hand, the emergence of wireless sensors and networks (WSNs) provides an excellent opportunity to finally realize the *in-situ*, real-time and on-line corrosion monitoring [22–27]. WSNs enable the engineers to remotely collect and transmit the electrochemical data through wireless channels, even under the most severe conditions, avoiding a large amount of wiring, and thus saving large amounts of labor and financial resources. In the future, WSNs should be able to support the corrosion monitoring and control as the hardware platform based on the concept of the Internet of Things. Considering the great advantages of WSNs and electrochemical emission spectra, we first attempted to design a novel pitting corrosion-detecting sensor device and a feasible, effective wireless monitoring platform to monitor the pitting corrosion at the nucleation stage; next, we propose a wavelet-based algorithm for analyzing the electrochemical emission data; finally, we evaluate the feasibility and efficiency of the wireless corrosion monitoring system working on RC panel test-beds.

## 2. Wireless Corrosion Monitoring Sensor and Network

Compared with the traditional corrosion monitoring approaches, wireless technology-based monitoring is very efficient in terms of cost, convenience, and human-operation overhead, and thus has attracted much attention in the past few years. In this work, we design a WSN to monitor the corrosion of RC structures. The advantages of our WSN-based test-beds are as follows. First, the designed corrosion-detecting device can be built using commercial off-the-shelf wireless motes, such as the Crossbow Mica mote and the Berkeley Telosb mote, so the hardware costs are very low, and the network is easily extended. Second, once the corrosion-detecting device is attached to the wireless mote, we can control the sampling rate by dynamically setting its parameters in an over-the-air fashion.

### 2.1. Corrosion-Detecting Sensor Mote Design

In designing the corrosion sensing mote platform, we have to address the following three problems: (1) what sensing device will be used in corrosion signal detection, (2) which kind of wireless mote is appropriate for our objectives in terms of cost and extensibility, and (3) how to connect the corrosion-detecting device to the wireless mote. We will present the designed corrosion-detecting devices in the later sections. Next, we will introduce the MicaZ mote in use and the designed circuit connecting the corrosion-detecting device with the MicaZ mote.

**Figure 1.**
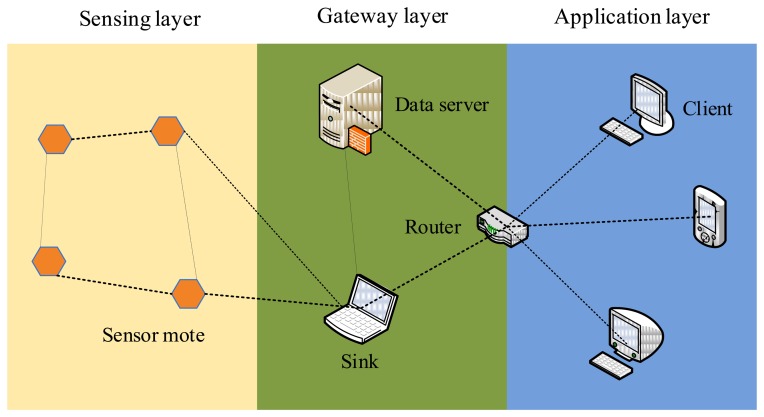
The architecture of the wireless corrosion monitoring system.

As a general wireless embedded platform, the MicaZ mote can not only measure 0–3 V with 0.01 mV resolution, but also be extended to monitor diverse other signals (such as temperature, humidity and vibration, *etc.*), so we used the MicaZ mote in our test-bed to support a closer monitoring of corrosion of RC structures. In our design, we consider mainly two issues related to the WSN platform: one is how to connect the corrosion-detection device to the MicaZ mote, and the other one is how to implement a wireless communication algorithm to effectively collect the corrosion data. Figure 1 shows the architecture of our test-bed. The sensing layer consists of a set of sensor motes, which will self-organize into a wireless network. The laptop, data server, or other Internet-accessing devices belong to the gateway layer which is responsible for collecting all the data sent by the sensing layer. The back-end application layer provides a visual interface for users as well as domain experts. As long as users can access the Internet, they can browse the data and the corresponding analysis results, or even download them.

**Figure 2.**
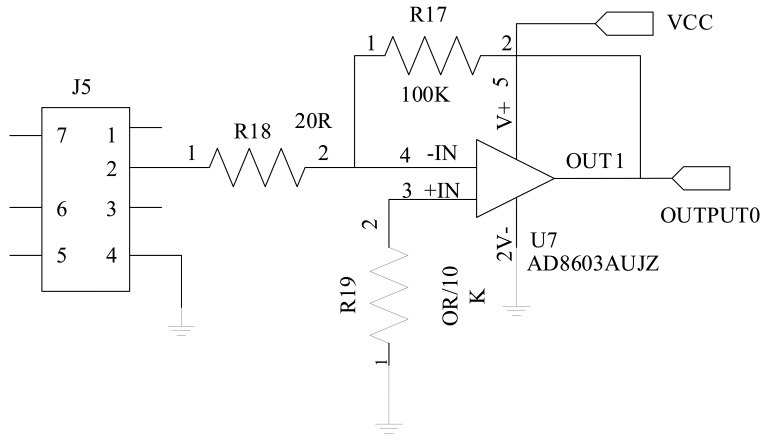
Schematic of the connecting circuit for the wireless corrosion sensor.

Even though the MicaZ mote is fitted with a 51-pin connector for external sensing devices, it cannot directly read the signal output of our corrosion-detection devices. This is because MicaZ's analog-to-digital conversion (ADC) interface (channel) cannot directly measure the current. However, the corrosion current is what we are very concerned about in corrosion evaluation. We therefore designed a connecting circuit between the corrosion-detecting device and the MicaZ mote, and the circuit functions by transforming the current output of the corrosion events into a voltage that can be measured by the MicaZ. We have to map each voltage reading of ADC channel of MicaZ into a current reading. Figure 2 shows the kernel part of the circuit [28]. The accuracies of the potential and current measurements are 10^−5^ V and 10^−7^ A, respectively.

The intermediate circuit uses an Analog Devices OP-amp of AD8603AUJZ, which is a low-power, rail-to-rail amplifier with the working current less of than 50 μA. Since the circuit needs a very low power level, the total power requirement of the circuit and the MicaZ mote can be kept within 600 μA, effectively prolonging the system lifetime. Additionally, an array of resistors is used to idey the corrosion potentials, and this makes the circuit be able to measure at the 0.1 μA level, the precision of which is completely sufficient for our scenario.

### 2.2. Wireless Corrosion-Monitoring Network

**Figure 3.**
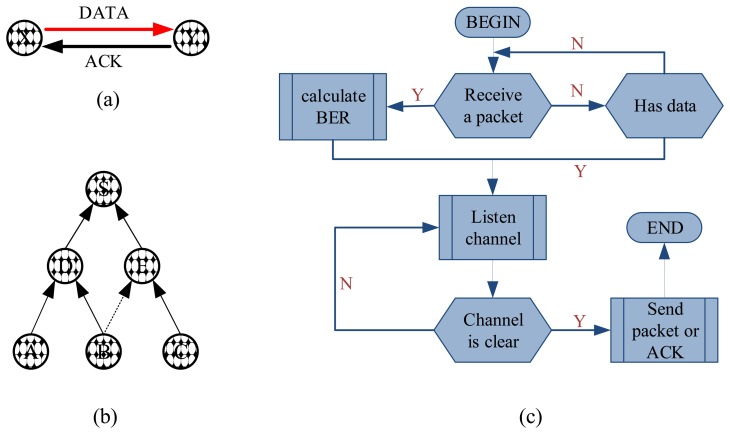
Routing algorithm for the wireless corrosion monitoring network.

For the corrosion-monitoring sensor mote platform, another challenge is how to collect the corrosion data from all the motes with as little human intervention as possible. Using the TinyOS operating system for MicaZ and the Xserve communication framework, we propose a many-to-one routing protocol, to transport data from all sensor motes to a central mote (also called sink). The sink, storing all the corrosion data, can display it visually, and domain scientists can then well understand and predict the progress of the corrosion process. In the proposed routing algorithm, the sensor motes are organized into a multi-hop network which can work in the following two ways: (1) the sink broadcasts a query message, and the motes receiving this message send data back to the sink, and (2) all the motes report their data to the sink regularly, e.g., once per day. In our experiments, we use the latter one. To guarantee the reliability of the data transmission, two schemes are adopted in our work. First, as shown in Figure 3a, we employ the Acknowledgement (ACK) scheme of the IEEE 802.15.4 protocol [29] to improve the link quality with multiple transmissions. Second, a mote always chooses the mote with maximal link quality as its forwarder (the link quality is generally measured by the reception ratio of packets over the link). For example, the mote *B* in Figure 3b has two possible forwarders, *D* and *E*, but it will select *D* as the next hop if the link to *D* has higher quality. Based on the CC2420 radio chip used by MicaZ, the link quality can be calculated at the hardware level as the bit error rate (BER) value and consequently, needs no extra codes. Even though the link quality is obtained at the receiver, it can be piggy-backed by the ACK messages from the receiver to the transmitter. By doing so, the transmitter has information about the qualities of links to all the potential forwarders. Figure 3c presents the detailed control logics of our routing algorithm. Note that these control programs can be updated over the air, even if they have been deployed in motes. Such a reprogramming method without recycling motes facilitates the deployment of the new monitoring tasks. The experiments results show that only two packets are lost due to link failure within two hours in our routing scheme.

## 3. Energy Distribution Algorithm of EES in Wavelet Domain

**Figure 4.**
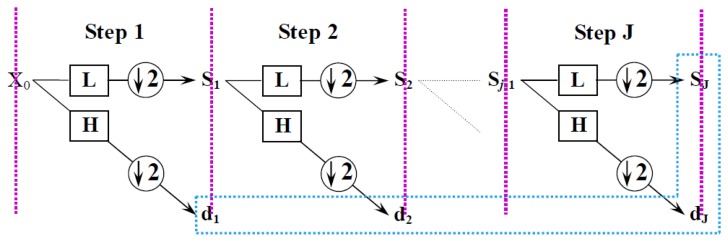
Schematic of the fast wavelet transform algorithm.

The fast wavelet transform algorithm for electrochemical emission signals is illustrated in Figure 4. The energy accumulated in the subspace $U_{j}^{n}\left(n\in Z_{+}\right)$ can be calculated by:

(1) $$E_{j}^{n}=\sum_{k}{\left(d_{j,k}^{n}\psi_{j,k}^{n}\left(t\right)\right)}^{2}$$

(2) $$E_{j}^{\prime n}=\sum_{k}{\left(f_{j}^{n}\left(t\right)\right)}^{2}$$

where $d_{j,k}^{n}$, $\psi_{j,k}^{n}\left(t\right)$ and $f_{j}^{n}\left(t\right)$ are the wavelet coefficients, mother wavelets and the reconstructed signal in $U_{j}^{n}\left(n\in Z_{+}\right)\cdot j$ is the depth of wavelet decomposition, and *j* ∈ [1, *J*]. As the wavelet basis is a complete orthogonal basis, $E_{j}^{n}$ equals $E_{j}^{\prime n}$.

To eliminate the influence of the trend in the electrochemical emission signal, the decomposed signal $D_{J,k}^{n}$ is removed in the reconstructed data in *J* level to dimensionlessly normalize the energy accumulated in $U_{j}^{n}\left(n\in Z_{+}\right)$ as follows:

(3) $$\bar{E_{j}^{n}}=\frac{E_{j}^{n}}{\sum_{n}E_{j}^{n}}$$

Then, we can obtain the energy distribution of the discrete electrochemical emission signals *x_n_*(*t*), *n* = 1, 2, …, *N* on each scale as follows:

(4) $$\bar{E}=\left[\bar{E_{1}},\bar{E_{2}},\cdots ,\bar{E_{J}}\right]$$

where $\bar{E_{1}}$, $\bar{E_{2}}$ and $\bar{E_{J}}$ are the dimensionless normalized energy accumulated on the 1, 2 and *J* scales, respectively.

## 4. Experiments

Two kinds of typical RC panels which represent the passive and active state were used to verify the effectiveness of the proposed WSN for corrosion monitoring. One RC panel was poured with plain concrete, and the other was formed with concrete containing 3.5% NaCl by weight of mixing water. The mix proportions of C30 concrete is listed in Table 1. The cement is P.O.42.5 Portland cement (Yatai Group Harbin Cement Co., Ltd., Harbin, China). The content of polycarboxylate water reducing agent UNF-5 (Kanghua Chemical Co., Ltd., Jilin, China) is 1.0% by the weight of cement. After curing the panels for 2 months with 17 ± 3 °C and 70% ± 10% relative humidity (RH) condition as controlled by a SEEKE SP-30AT6DT system (Jiedi Electronic Equipment Manufacturing Co., Ltd., Nanjing, China), the WSN-based corrosion monitoring tests were performed.

**Figure 5.**
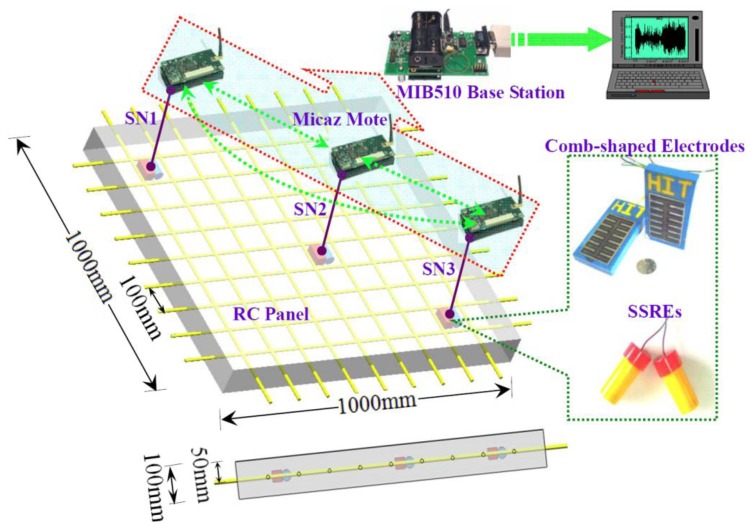
The schematic plan of the wireless corrosion monitoring system for RC panels.

Figure 5 illustrates the geometry of the RC panels and the distribution of the sensing motes. To recycle the MicaZ motes in the future, the sensing elements including the comb-shaped electrodes and the solid-state reference electrodes (SSREs) prepared by electron-beam vapor deposition are directly embedded in the concrete surrounded by the steel-bar mesh, and these electrodes are wired with the MicaZ motes installed on the surface of the reinforced concrete panels.

The wireless mote entitled “SN0” is mounted on the plain RC panel to monitor the electrochemical emission data as the reinforcing steel is passivated. The other wireless motes entitled “SN1”, “SN2” and “SN3” are connected sequentially to the corresponding sensing elements embedded in the RC panel with 3.5% NaCl to monitor the corrosion status in the active state. It must keep in mind that the sensing elements and the MicaZ motes could be integrated and implanted into the concrete in practical engineering, and the power could be also supplied by the energy scavenged from wind, solar, concrete batteries or the electrochemical reactions happening during the corrosion process. The components of the comb-shaped electrode here include one graphite counter electrode and one Q235 carbon steel working electrode. The solid-state reference used as the baseline to monitor the change of the potential of the working electrode is developed based on NiFe_2_O_4_ electrochemical functional materials [30]. Its balance potential is −238 mV ± 5.0 mV (*vs.* Ag/AgCl/KCl_sat)_ in the simulated concrete pore solution (SCPS) at 25 °C. The sampling frequency of the electrochemical emission data is set to be 2 Hz.

## 5. Results and Discussion

**Figure 6.**
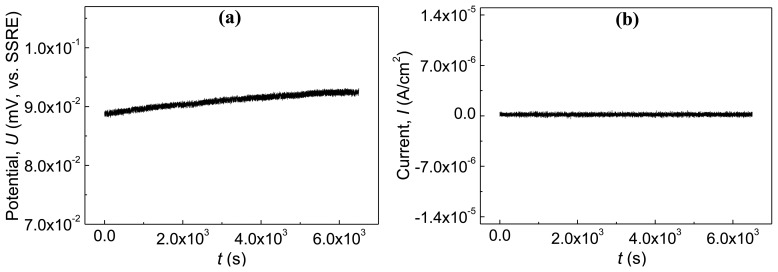
Electrochemical emission data of the passive state reinforced concrete panel. (**a**) and (**b**) are the electrochemical potential emission and the electrochemical current emission of the wireless mote SN0, respectively.

The electrochemical emission signals collected by the wireless corrosion monitoring sensor SN0 of the passive state reinforced concrete panel, including the potential and the current data, are presented in Figure 6a,b, respectively. According to Figure 6a, the potential emission is within a narrow band whose amplitude is no more than 3 mV (*vs.* SSRE). Besides, the current emission also shows a narrow-band-like distribution, and the amplitude is no more than 2 × 10^−7^ A/cm^2^. Which is close to the resolution of the wireless mote itself. The signal could be caused by the white noise of the acquisition system implanted in MicaZ mote or the passive steel/concrete system itself. These results indicate that the amplitude of potential or current emission of the reinforcing steel at passive state is very low, and the high pH value of the hydrated products of cement provides a safe environment to keep the r steel bar immune to chloride ions. Also, the wireless mote SN0 obtains the effective data correctly.

**Figure 7.**
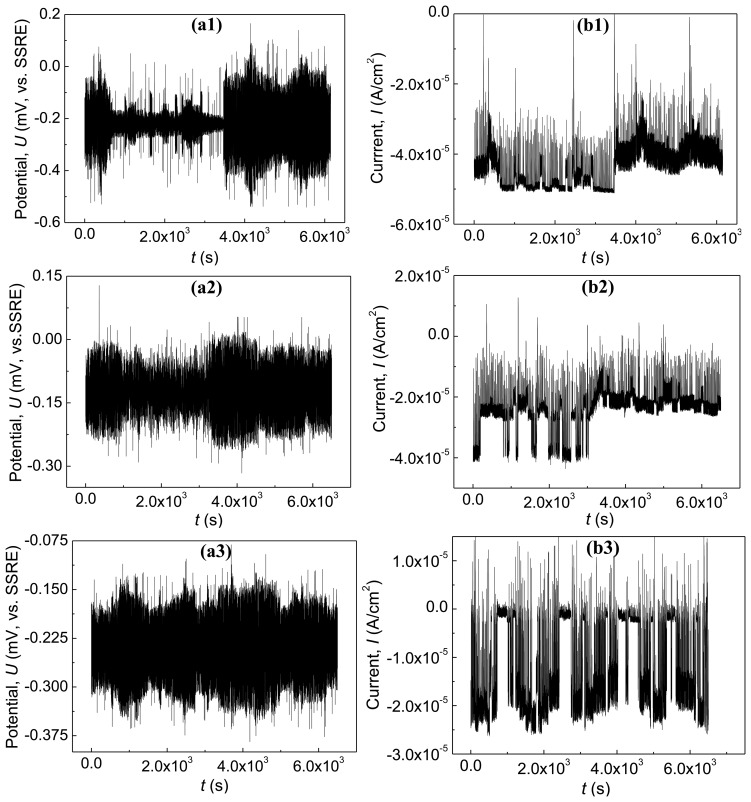
The electrochemical emission data of the active state RC panel. (**a1**), (**a2**), (**a3**) and (**b1**), (**b2**), (**b3**) are the electrochemical potential emission and electrochemical current emission of the wireless motes SN1, SN2 and SN3 located along the diagonal line of the RC panel in Figure 6, respectively.

Figure 7 exhibits the potential and the current emissions of the wireless motes SN1, SN2 and SN3 located along the diagonal line of the RC panel in Figure 5 with 3.5% NaCl solution. Lots of potential and current transients appear in the data. The amplitude of the potential emission transients and current emission transients achieve even hundreds of millivolts (*vs.* SSRE) and several of 10^−4^ A/cm^2^, respectively.

The typical attenuation life of electrochemical emission ranges from tens of seconds to a few hundreds of seconds. Note that the pitting corrosion presents stochastically on the surface of the working electrode. Generally, the pitting corrosion process of the steel bar in Cl^−^ solution includes four steps as follows: the adsorption of Cl^−^, the nucleation of metastable pits, collapse of the metastable pit and the formation of the new pit, and the formation and development of the stable pit. The measured current embodies the superimposed effect of the pitting process. This process simultaneously includes the nucleation of the metastable pit and the repassivation processes. During the developing stage of the pitting corrosion caused by Cl^−^, two of the typical events emerge on the surface of the specimen: one part is the passivating film and the other part is the active pits. In the later part, the active steel directly contacts with solution, and then the anode current becomes much larger. As such a large current flows through the solution in the pit, there exists a large voltage drop. According to the point defect model [31], the aggressive anion Cl^−^ is able to enhance the flux of cation vacancies through the barrier layer during the pitting corrosion process. With the favorable conditions (voltage, pH, [Cl^−^]), vacancy condensation will occur at the metal/barrier layer interface, and hence passivity breakdown will is confirmed, so the fresh surface of the metal will generate an electrochemical potential and current transients. We can also see that the wireless corrosion monitoring sensors and networks could effectively capture and transmit these electrochemical emission signals.

**Figure 8.**
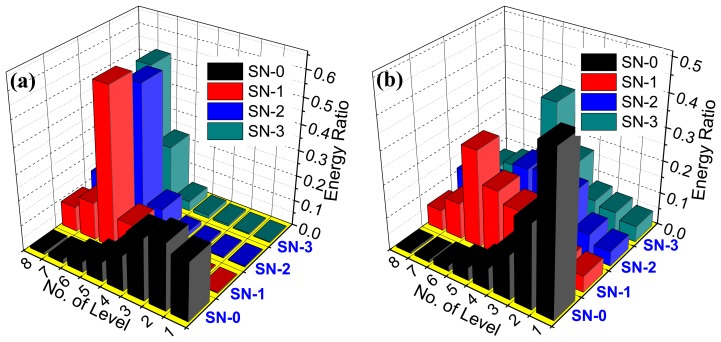
Energy distribution plots of the electrochemical emission signals in wavelet domain. (**a**) and (**b**) are the energy distribution plots of the electrochemical potential emission and electrochemical current emission, respectively.

To idey the pitting corrosion qualitatively, the energy distribution algorithm of the electrochemical emission signal in wavelet domain described in Section 3 of this paper is applied to analyze the potential and current emission data in Figures 6 and 7. The orthogonal Sym4 wavelet is utilized to decompose the electrochemical emission data in depth of 8-level, and the approximation signal in 8-level is removed as the harmful trend. Then, the decomposed signal in each level is reconstructed and the energy distribution plots are obtained based on the Equation (3). Figure 8a,b illustrates the energy ratio on each level of the potential and current emission data, respectively. According to Figure 8a, the energy of potential emission data collected by SN0 at passive sate accumulates on the level 1–3. The frequency of these parts between 1 and 2–3 Hz is much higher than that of other parts. However, the energy transfers to level 5–8 as the pitting corrosion appears. The energy condensed on these levels is no less than 80% of the total energy. The frequency of the levels from 5 to 8 is no more than 2–5 Hz. The condition of electrochemical current emission in Figure 8b is similar with that of the electrochemical potential emission in Figure 8a. According to the change of the energy distribution on the different crystal, the pitting corrosion is verified. The energy which is distributed on the level 5–8 is the intrinsic characteristics of pitting corrosion of the reinforcing steel in concrete. The energy distribution plot of the electrochemical emission signal can be applied as the benchmark to qualitatively idey the presence of the pitting corrosion. Actually, the *in-situ*, real-time and on-line corrosion monitoring information of the electrochemical emission spectrum could be applied to drive the 3-D cellular automata to quantitatively predict the development of the corrosion pit. Considering the aim of this paper, we do not discuss this point in detail here.

## 6. Conclusions

We have employed a WSN to realize a corrosion monitoring platform, which can acquire electrochemical emission signals during the pitting corrosion process, in an *in-situ*, real-time, and reliable way. Additionally, we have designed a wavelet-based algorithm to characterize the corrosion process of the RC structures, with the corrosion data collected by the WSN platform. We find that the amplitudes of the electrochemical current and potential emission are a few millivolts (*vs.* SSRE) and 10^−7^ A/cm^2^ degree in the passive state of the reinforcing steel, respectively. However, they increase to several hundreds of millivolts (*vs.* SSRE) and 10^−4^ A/cm^2^ degree in the active state. In particular, the energy distribution plot of the electrochemical emission signal returned by the proposed wavelet algorithm can serve as the benchmark to qualitatively verify the presence of pitting corrosion.

## Figures and Tables

**Table 1.** C30 concrete mixture proportions (units: kg/m^3^).

**Components**	**Cement**	**Aggregate**	**Sand**	**Water**	**NaCl**	**UNF-5**
Passive	390	800	1035	180	0	3.9
Active	390	800	1035	180	6.3	3.9

## References

[1] Hietpas K., Ervin B., Banasiak J., Pointer D., Kuchma D.A., Reis H., Bernhard J.T. (2005). Ultrasonics and electromagnetics for a wireless corrosion sensing system embedded in structural concrete. Smart Struct. Syst..

[2] Bertolini L., Elsener B., Pedeferri P. (2004). Corrosion of Steel in Concrete-Prevention, Diagnosis, Repair.

[3] Elsener B. (2000). Corrosion of steel in concrete. Mater. Sci. Technol..

[4] Catbas F.N., Shah M., Burkett J. (2004). Challenges in structural health monitoring. The 4th International Workshop on Structural Control.

[5] Li H.N., Li D.S., Song G.B. (2004). Recent applications of fiber optic sensors to health monitoring in civil engineering. Eng. Struct..

[6] Huang S.F., Ye Z.M., Hua Y.L., Jun C., Lua L.C., Cheng X. (2007). Effect of forming pressures on electric properties of piezoelectric ceramic/sulphoaluminate cement composites. Compos. Sci. Technol..

[7] Song G.B., Gu H.C., Mo Y.L., Hsu T.T.C., Dhonde H. (2007). Concrete structural health monitoring using embedded piezoceramic transducers. Smart Mater. Struct..

[8] Song G.B., Gu H.C., Mo Y.L. (2008). Smart aggregates: Multi-functional sensors for concrete structures—A tutorial and a review. Smart Mater. Struct..

[9] Qiao G.F., Hong Y., Sun G.D., Yang O. (2013). Corrosion energy: A novel source to power the wireless sensor. IEEE Sens. J..

[10] Han B.G., Yu Y., Han B.Z., Ou J.P. (2008). Development of a wireless stress/strain measurement system integrated with pressure-sensitive nickel powder-filled cement-based sensors. Sens. Actuators A Phys..

[11] Li H., Xiao H.G., Ou J.P. (2008). Electrical property of cement-based composites filled with carbon black under long-term wet and loading condition. Compos. Sci. Technol..

[12] Ou J.P., Li H. The State-of-the-Art and Practice of Structural Health Monitoring for Civil Infrastructures in the Mainland of China.

[13] Ou J.P., Li H. (2010). Structural health monitoring in mainland China: Review and future trends. Struct. Health Monit..

[14] Achenbach J.D. (2009). Structural health monitoring—What is the prescription?. Mech. Res. Commun..

[15] Sun Z., Mansfeld F. (1999). Localization index obtained from electrochemical noise analysis. Corrosion.

[16] Qiao G.F., Xiao H.G., Sun G.D. (2011). Ideication of the reinforcing steel's corrosion state in RC beams based on electrochemical sensor. Sens. Rev..

[17] Smulko J.M., Darowicki K., Zieliñski A. (2007). On electrochemical noise analysis for monitoring of uniform corrosion rate. IEEE Trans. Instrum. Meas..

[18] Aballe A., Bethencourt M., Botana F.J., Marcos M., Sánchez-Amaya J.M. (2001). Use of wavelets to study electrochemical noise transients. Electrochim. Acta.

[19] Montes-García P., Castellanos F., Vásquez-Feijoo J.A. (2010). Assessing corrosion risk in reinforced concrete using wavelets. Corros. Sci..

[20] Muniandy S.V., Chew W.X., Kan C.S. (2011). Muractal modelling of electrochemical noise in corrosion of carbon steel. Corros. Sci..

[21] Sun G.D., Qiao G.F., Xu B. (2011). Corrosion monitoring sensor networks with energy harvesting. IEEE Sens. J..

[22] Qiao G.F., Ou J.P. (2007). Corrosion monitoring of reinforcing steel in cement mortar by EIS and ENA. Electrochim. Acta.

[23] Park C., Xie Q., Chou H. DuraNode: Wireless Networked Sensor for Structural Health Monitoring.

[24] Paek J., Chintalapudi K., Caffrey J., Govindan R., Masri S. A Wireless Sensor Network for Structural Health Monitoring: Performance and Experience.

[25] Park G., Farrar C.R., Todd M.D. (2007). Energy Harvesting for Structural Health Monitoring Sensor Networks.

[26] Wu J., Wu W.C. (2010). Study on wireless sensing for monitoring the corrosion of reinforcement in concrete structures. Measurement.

[27] Qiao G.F., Liu T.J., Dai J.H., Hong Y., Wan J. (2012). Qualitative and quantitative sensors based on electrochemical techniques for the corrosion assessment of RC panels. IEEE Sens. J..

[28] Qiao G.F., Sun G.D., Hong Y., Qiu Y., Ou J.P. (2011). Remote corrosion monitoring of the RC structures using the electrochemical wireless energy-harvesting sensors and networks. NDT&E Int..

[29] IEEE 802.15 WPAN™ Task Group 4 (TG4). http://www.ieee802.org/15/pub/TG4.html.

[30] Qiao G.F., Hong Y., Song G.P., Li H., Ou J.P. (2012). Electrochemical characterization of the solid-state reference electrode based on NiFe_2_O_4_ film for the corrosion monitoring of RC structures. Sens. Actuators B Chem..

[31] Macdonald D., Marx B.M. (2004). Development of advanced electrochemical emission spectroscopy for monitoring corrosion in simulated DOE liquid waste.

